# Self-Organized TiO_2_–MnO_2_ Nanotube Arrays for Efficient Photocatalytic Degradation of Toluene

**DOI:** 10.3390/molecules22040564

**Published:** 2017-03-31

**Authors:** María C. Nevárez-Martínez, Marek P. Kobylański, Paweł Mazierski, Jolanta Wółkiewicz, Grzegorz Trykowski, Anna Malankowska, Magda Kozak, Patricio J. Espinoza-Montero, Adriana Zaleska-Medynska

**Affiliations:** 1 Facultad de Ingeniería Química y Agroindustria, Escuela Politécnica Nacional, Ladrón de Guevara E11-253, P.O. Box 17-01-2759, Quito 170525, Ecuador; ma.cristina.nevarez@gmail.com; 2 Centro de Investigación y Control Ambiental “CICAM”, Departamento de Ingeniería Civil y Ambiental, Facultad de Ingeniería Civil y Ambiental, Escuela Politécnica Nacional, Ladrón de Guevara E11-253, P.O. Box 17-01-2759, Quito 170525, Ecuador; patricio.espinoza@epn.edu.ec; 3 Department of Environmental Technology, Faculty of Chemistry, University of Gdansk, 80-308 Gdansk, Poland; marek.kobylanski@phdstud.ug.edu.pl (M.P.K.); anna.malankowska@ug.edu.pl (A.M.); magda.kozak@ug.edu.pl (M.K.); 4 Faculty of Chemistry, Nicolaus Copernicus University, 87-100 Torun, Poland; jolanta.wolkiewicz@umk.pl (J.W.); tryki@umk.pl (G.T.)

**Keywords:** TiO_2_–MnO_2_ nanotubes, visible light induced photocatalysis, alloys, toluene degradation, anodization

## Abstract

Vertically oriented, self-organized TiO_2_–MnO_2_ nanotube arrays were successfully obtained by one-step anodic oxidation of Ti–Mn alloys in an ethylene glycol-based electrolyte. The as-prepared samples were characterized by scanning electron microscopy (SEM), energy-dispersive X-ray spectroscopy (EDX), UV-Vis absorption, photoluminescence spectroscopy, X-ray diffraction (XRD), and micro-Raman spectroscopy. The effect of the applied potential (30–50 V), manganese content in the alloy (5–15 wt. %) and water content in the electrolyte (2–10 vol. %) on the morphology and photocatalytic properties was investigated for the first time. The photoactivity was assessed in the toluene removal reaction under visible light, using low-powered LEDs as an irradiation source (λ_max_ = 465 nm). Morphology analysis showed that samples consisted of auto-aligned nanotubes over the surface of the alloy, their dimensions were: diameter = 76–118 nm, length = 1.0–3.4 μm and wall thickness = 8–11 nm. It was found that the increase in the applied potential led to increase the dimensions while the increase in the content of manganese in the alloy brought to shorter nanotubes. Notably, all samples were photoactive under the influence of visible light and the highest degradation achieved after 60 min of irradiation was 43%. The excitation mechanism of TiO_2_–MnO_2_ NTs under visible light was presented, pointing out the importance of MnO_2_ species for the generation of e^−^ and h^+^.

## 1. Introduction

TiO_2_-based photocatalysis is an effective technique for pollutant removal from both gas and liquid phase [1,2,3,4,5,6,7]. In fact, applications of TiO_2_ are not limited only to photodegradation reactions, but it offers the facility to drive many others such as organic synthesis [8], water splitting [9,10], disinfection [11], CO_2_ reduction [10,12], self-cleaning or antimicrobial surfaces [13,14], and dye-sensitized solar cells [10,15,16]. Due to the environmentally-friendly nature of TiO_2_, its chemical and biological inertness, low cost, availability, and excellent photoactivity, this semiconductor material has become of great interest [17]. Nevertheless, some drawbacks as the rapid charge recombination of the photogenerated electrons and holes, and the wide bandgap (3.0 eV for rutile and 3.2 eV for anatase), which restricts photoabsorption to only ultraviolet region (ca. 5% of solar spectrum), need to be overcome in order to extend the practical application of TiO_2_ photocatalysts for solar or interior light driven photoreactions at large scale [18].

Bandgap engineering in addition to tuning strategies have been studied over last decades with a common aim: shifting the absorption wavelength range of TiO_2_ to the visible region. Since 1980s, TiO_2_ has been modified by platinization [19,20,21]. So far, numerous approaches as ion (either cation or anion) doping [22,23,24,25], coupling with a narrower-bandgap semiconductor [26], with noble metals [27,28], with either organic or inorganic dyes [29] have been presented by a large number of research groups. Synthesizing composites with oxide semiconductors has become a promising way to enhance the photoactivity of TiO_2_ by promoting the absorption of visible light and inhibiting the fast recombination of charge carriers [18,30]. Recent studies have focused on TiO_2_–MnO_2_ system due to the MnO_2_ features as non-toxicity and earth abundance. These composites have been used mainly for capacitance applications [31,32], and despite the narrow bandgap of MnO_2_ (0.26–2.7 eV), which could allow the absorption of visible and theoretically even infrared light [33,34,35,36,37,38], there exist just few reports in literature about the application of this system in photocatalysis. Xue, et al. [39] synthesized mesoporous MnO_2_/TiO_2_ nanocomposite, photoactive for the visible light-driven degradation of MB. They attributed the improved photocatalytic efficiency to the effective separation of photogenerated electrons and holes.

However, the industrial usage of photocatalysts is still in need of improvements to maximize the overall efficiency which also depends on mass and charge transfer processes. Therefore, TiO_2_ nanostructures like zero-dimensional (nanoparticles), one-dimensional (nanowires, rods, and tubes), two dimensional (layers and sheets), and three dimensional (hierarchical spheres) have been widely synthesized, and used [40]. Since the discovery of carbon nanotubes in 1991 by Iijima [41], 1D morphologies as nanotubes (NTs) have become attractive materials due to the efficient separation of charge carriers, shape selectivity in chemical processes, high surface area to volume ratio, high electron mobility, mechanical strength [40,42], and high photoactivity in air purification [43]. Many approaches as sol-gel, template assisted, hydro/solvothermal and electrochemical have achieved to prepare TiO_2_ NTs. Among these techniques, the electrochemical anodization of a suitable metal or alloy is the simplest, cheapest, and the most direct to grow self-highly-organized-nanotube arrays under specific electrochemical conditions which permit to control the properties of the fabricated NTs [44]. It was reported previously that MnO_2_-TiO_2_ NTs composite could be successfully formed by one-step anodic oxidation of Ti–Mn alloy [22,37]. Mohapatra, et al. [45] synthesized ordered arrays of mixed oxide NTs by anodization of Ti/Mn alloys, under ultrasonication in the presence of a fluoride-containing ethylene glycol solution. They pointed out that before calcination, the as-formed NTs showed a stoichiometry of (Ti,Mn)O_2_, while annealing at 500 °C resulted in formation of nanotubes composed of anatase and rutile phases of TiO_2_ and Mn_2_O_3_. Ning, Wang, Yu, Li, and Zhao [32] electrochemically prepared mixed oxide NTs from Ti–Mn alloys which showed enhanced capacitive properties compared with those of pristine TiO_2_ NTs. 

Herein, this work aims to anodically grow TiO_2_–MnO_2_ NTs in a fluoride-containing ethylene glycol-based electrolyte, and their application in the photodegradation of a model gaseous pollutant. According to our best knowledge, photocatalytic properties of nanotubes made of titania and manganese oxide mixtures have been investigated in this work for the first time. Moreover, parameters as the applied voltage (30–50 V), manganese content in the alloy (5–15 wt. %), and water content (2–10 vol. %) in the electrolyte have been also studied for the first time to analyze their effect on the morphology and photoactivity of the obtained NT arrays. Photodegradation tests in the gas phase were conducted with toluene as the model pollutant, and a possible mechanism of visible-light driven decomposition over the TiO_2_–MnO_2_ NTs was proposed as well.

## 2. Results and Discussion

### 2.1. Morphology and Growth Mechanism

**Figure 1:**
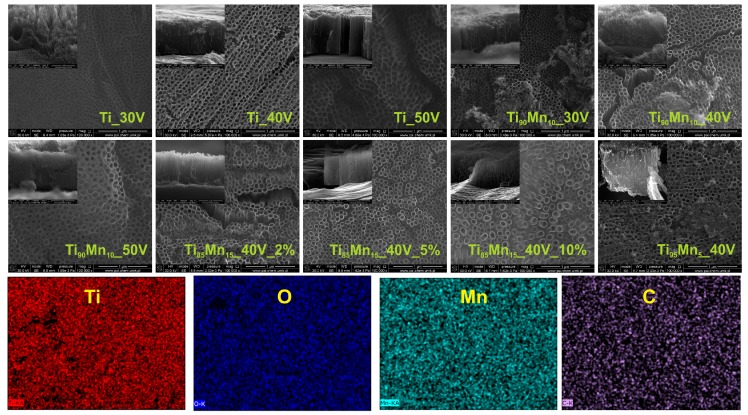
Top-view and cross-sectional SEM images of pristine TiO_2_ and TiO_2_–MnO_2_ NTs (the effect of applied voltage, manganese content in the Mn/Ti alloy, and water content in the electrolyte on the morphology of formed nanotubes) and EDX mapping of the Ti_90_Mn_10__30V sample.

One-step anodization processes were conducted for 60 min to synthesize pristine TiO_2_ and TiO_2_–MnO_2_ nanotube layers from technical grade Ti sheets and Ti–Mn alloys under specific conditions, which are summarized in Table 1. SEM technique was used to analyze the effect of the applied voltage (30, 40 and 50 V), manganese content in the alloy (5, 10 and 15 wt. %) and water content in the electrolyte (2, 5 and 10 vol. %) on the morphology of the as-prepared samples. Figure 1 shows the top and cross-sectional SEM images which indicate that all synthesized nanotubes were uniform and vertically oriented. Pristine TiO_2_ NTs presented smooth and uniform walls while TiO_2_–MnO_2_ NTs had ripples on their walls, which was also observed by Mohapatra, et al. [45] in samples anodized from Ti-8Mn alloys. It is well known that the dimensions of the nanotubes can be easily tuned by changing the preparation parameters [25]. Length and diameter increased with increasing the applied voltage, starting from d = 81 ± 9 nm and l = 1.5 ± 0.1 μm (Ti_30V); and reaching values of d = 120 ± 12 nm and l = 16.2 ± 0.2 μm (Ti_50V) for pristine TiO_2_ NTs. The influence of anodization voltage was studied keeping constant the manganese content in the alloy (10 wt. %) and the water content in the electrolyte solution (2 vol. %). This way, dimensions of samples synthesized from Ti_90_Mn_10_ alloy also were bigger as the applied potential was higher, starting from d = 76 ± 9 nm and l = 1.0 ± 0.1 μm (Ti_90_Mn_10__30V) and rising to d = 118 ± 4 nm and l = 2.8 ± 0.1 μm (Ti_90_Mn_10__50V). Similar behavior of morphological results were reported by Macak, et al. [46]. They performed a systematic study of the factors influencing the two-step anodization of Ti foils in ammonium fluoride-containing glycerol/water mixtures. They prepared NT layers with diameters in the range of 20–300 nm for the potentials 2–40 V, while the thickness of the NT layers, tube length, was in the range of 150 nm up to 3 μm. This dependence of the dimensions, diameter, and length, with the applied potential is in well agreement with the present work. However, herein, the electrolyte media (EG-based) favored longer tubes in the case of pristine TiO_2_ NTs. Furthermore, a complementary discussion about the anodization voltage effect on the diameter of NT arrays has been reported by Macak, et al. [47]. They stated that, particularly for TiO_2_ NTs, the diameter strongly depends on the applied potential and electrolyte media, and consequently a wide variety of nanotube diameters can be obtained.

The samples fabricated at 40 V from Ti_85_Mn_15_ alloy in electrolytes with different water content (2–10 vol. %) reported smaller length (1.1–1.3 μm) than that of the analogous non-modified (Ti_40V, 5.0 ± 0.4 μm). As it was mentioned in previous works [44,46], the increase of water in the electrolyte, provoked the increase in the formation of ripples in the tube walls. The sample prepared from the alloy with 5 wt. % of Mn showed the longest modified nanotubes (3.4 ± 0.3 μm), presumably due to the low content of Mn in the alloy which allowed a better stabilization of the nanotube matrix by TiO_2_ species. Detailed information is displayed in Table 1. As it can be seen, all TiO_2_–MnO_2_ NTs were shorter, with smaller wall thickness than their pristine analog. Their length decreased with increasing the manganese content in the alloy. This could be attributed to the increase in the dissolution rate in phases with higher manganese content [32,45].

Table 1 also presents the results from EDX analysis which is in accordance with the composition of the alloys and no elements different from Ti, Mn, C, and O were found. Figure 1 presents also the EDX mapping of a selected sample where all elements are well dispersed and thus, there was not aggregation of Ti and Mn which guaranteed chemically homogeneous nanotube arrays.

**Figure 2:**
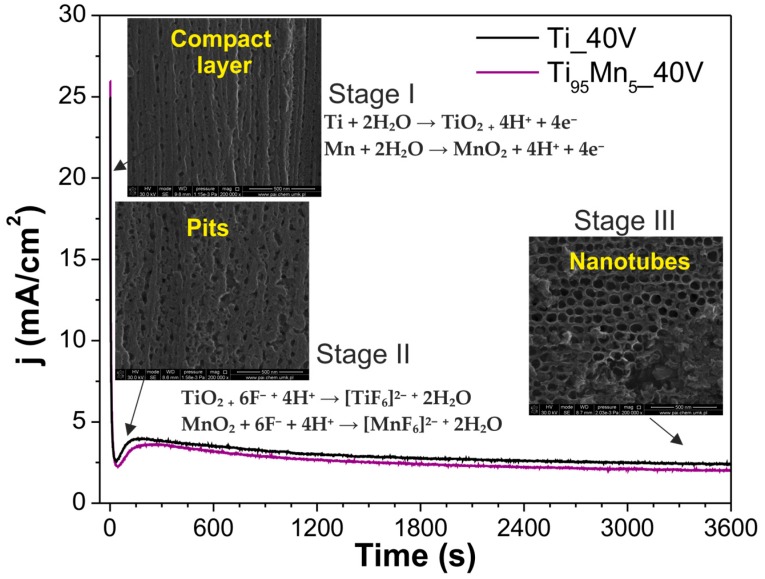
Proposed growth mechanism of TiO_2_–MnO_2_ NTs.

A possible growth mechanism was proposed in Figure 2, based on the obtained results from SEM images of the sample Ti_95_Mn_5__40V anodized during 4, 15 and 60 min and information provided in literature. It is possible to observe that the current density-time curves recorded for TiO_2_–MnO_2_ NTs resemble those corresponding to pristine TiO_2_ NTs. The characteristic exponential decay of current density during the first stage indicates the formation of the oxide layer composed of TiO_2_ and MnO_2_ [45], Progressively, the chemical etching induces the apparition of initial random pits in the mixed oxide layer due to its dissolution through the formation of the fluoride complexes [TiF_6_]^2−^ and [MnF_6_]^2−^ [48,49]. Consequently, the resistive field decreases, allowing the current density to increase along the second stage. Finally, throughout the third stage, an equilibrium is established between oxidation and chemical dissolution, leading to the self-organized nanotube growth under steady state conditions [47] allowing the auto-alignment of the nanotubes.

### 2.2. Structural Properties

**Figure 3:**
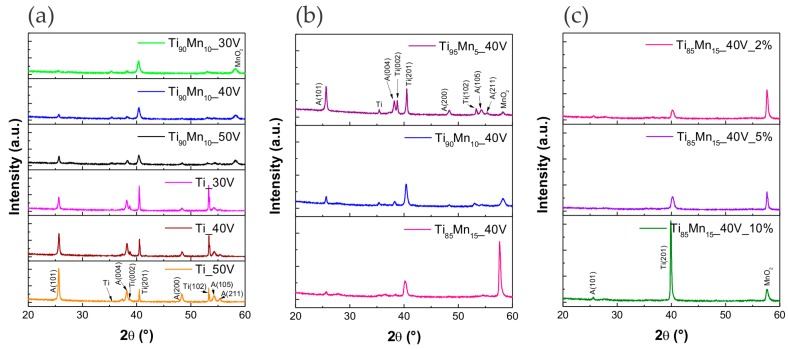
XRD spectra of pristine TiO_2_ and TiO_2_–MnO_2_ NTs. Effect of (**a**) anodization potential; (**b**) manganese content in the alloy; and (**c**) water content in the electrolyte.

The XRD patterns of the as-obtained NTs are presented in Figure 3. As it can be seen, obtained pristine and TiO_2_–MnO_2_ NTs consisted mainly of pure anatase TiO_2_, while the peaks of Ti came from Ti substrate. Five common planes of anatase were found, namely (101), (004), (200), (105) and (211). The intensity of anatase diffraction peaks increased with increasing the anodization potential as a result of thicker NT layer. It was possible to observe just one characteristic peak ascribed to MnO_2_ at about 58° [50,51]. The absence of any other band corresponding to the signature of MnO_2_ can be related to the small content and good dispersion of manganese oxide in the TiO_2_ NT layer, as it was mentioned in previous reports [32,39,52]. However, the constant diffraction peak positions indicate that the structure of TiO_2_ was not changed through the anodization of Ti–Mn alloy.

The calculated average crystallite size for pristine and modified TiO_2_ NTs is summarized in Table 1. The average crystallite size was calculated using the Scherrer equation, based on (101) diffraction peak. The largest crystallite size was observed for pristine TiO_2_ NTs and varied from 33 (30 V) to 38 nm (50 V). Among Ti–Mn series, crystallite sizes tended to be smaller than those of pristine TiO_2_ NTs. This can be correlated to the wall thickness, as mentioned above, wall thickness of TiO_2_–MnO_2_ NTs was smaller than that of pristine TiO_2_ NTs, thus there is less space to allow the growth of grain.

**Figure 4:**
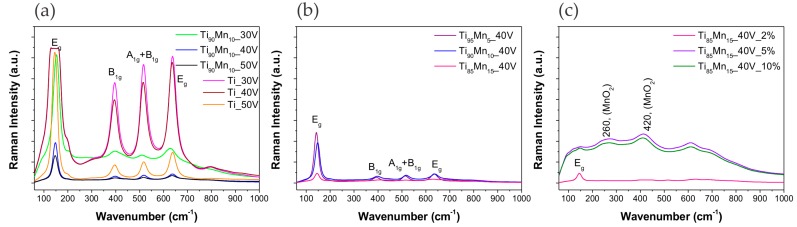
Raman spectra of pristine TiO_2_ and TiO_2_–MnO_2_ NTs. Effect of (**a**) anodization potential; (**b**) manganese content in the alloy; and (**c**) water content in the electrolyte.

To further analyze the structure of the synthesized photocatalysts, micro-Raman spectroscopy was performed using a 532 nm laser as excitation light. Figure 4 displays the recorded spectra of pristine TiO_2_ and TiO_2_–MnO_2_ NTs. As it can be seen, the spectra of the samples obtained from alloys with 5 and 10% of Mn mainly presented the signature peaks of anatase phase which are sharper in the spectra of pristine TiO_2_ NTs. These peaks at approximately 150, 396, 515, and 636 cm^−1^ can be attributed to the E_g_ (TiO_2_ symmetry), B_1g_ (O–Ti–O bending), A_1g_ + B_1g_ (T–O stretching), and E_g_ modes of anatase as it was exposed in previous reports [53]. The presence of MnO_2_ in these samples decreased the intensity and broadened the anatase bands. On the other hand, the characteristic peaks of MnO_2_ at around 521 and 644 cm^−1^, assigned to the stretching mode of octahedral MnO_6_ [54], overlapped the anatase peak at 636 cm^−1^ in the spectra of the samples prepared from Ti_85_Mn_15_ alloys, making it broaden to a range of 575–650 cm^−1^ [52]. These spectra also showed week bands at about 260 and 420 cm^−1^ originated from the bending modes of the metal–oxygen chain of Mn–O–Mn in the MnO_2_ octahedral lattice [55,56,57].

### 2.3. Optical Properties

**Figure 5:**
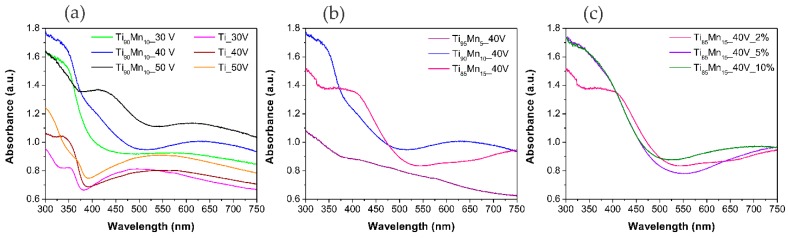
UV-Vis spectra of pristine TiO_2_ and TiO_2_–MnO_2_ NTs. Effect of (**a**) anodization potential; (**b**) manganese content in the alloy; and (**c**) water content in the electrolyte.

Figure 5 shows the absorption spectra of pristine TiO_2_ and TiO_2_–MnO_2_ NTs. All the samples synthesized from the Ti–Mn alloy exhibited absorption in the full visible range due to the presence of MnO_2_ as it was previously reported for TiO_2_ NTs coated by MnO_2_ [58]. The absorption band edge of pure TiO_2_ NTs at about 400 nm registered a red-shift at about 500 nm which is easier to appreciate in samples prepared from alloys with 15% of Mn. This was also observed in the case of mesoporous structured MnO_2_/TiO_2_ nanocomposites [39]. As it was stated by Ding, et al. [59], TiO_2_–MnO_2_ NTs could be used for solar-light driven photocatalysis owing to their absorption in the UV and visible region.

**Figure 6:**
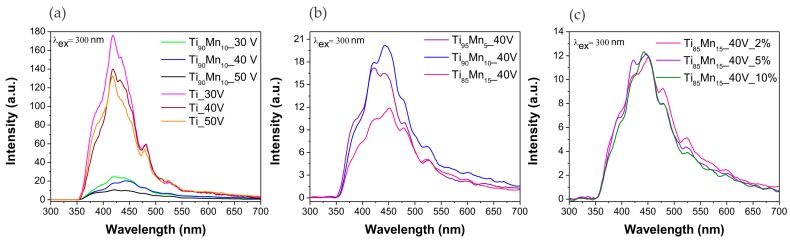
Photoluminescence spectra of pristine TiO_2_ and TiO_2_–MnO_2_ NTs. Effect of (**a**) anodization potential; (**b**) manganese content in the alloy; and (**c**) water content in the electrolyte.

Figure 6 shows photoluminescence (PL) spectra of both: pristine TiO_2_ and TiO_2_–MnO_2_ NTs. Four emission peaks were detected among all series of photocatalysts. First one, at approximately 420 nm can be ascribed to the existence of self-trapped excitons from TiO_6_^8−^octahedron, while the second and third peaks at 450 and 485 nm are associated with the presence of surface defects and oxygen vacancies. The last peak at approximately 525 nm is associated with radiative recombination of charge carriers [60,61].

### 2.4. Photocatalytic Performance

**Figure 7:**
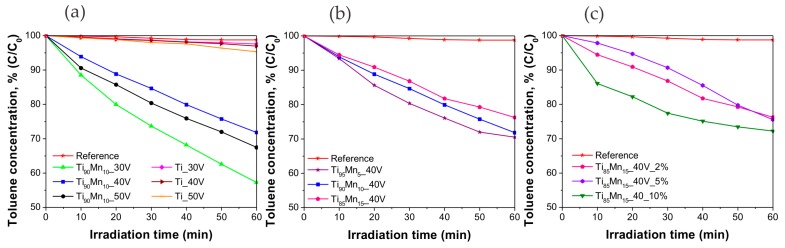
Photoactivity of pristine TiO_2_ and TiO_2_–MnO_2_ NTs in gas phase degradation of toluene under Vis light irradiation (λ_max_ = 465 nm). Effect of (**a**) applied voltage; (**b**) manganese content in the alloy, and (**c**) water content in the electrolyte.

The photoactivity of the prepared samples was tested in the visible-light-driven photodegradation of toluene (200 ppmv) from an air mixture. The irradiation source consisted of a LED array with λ_max_ = 465 nm. The effect of anodization voltage, manganese content in the alloy and water content in the electrolyte was systematically studied. Figure 7 presents the degradation curves in the presence of obtained NT photocatalysts and a reference curve in the absence of any photocatalyst, to test photolysis. It is clearly showed that in the reference curve, degradation was not achieved. Pristine TiO_2_ NTs exhibited insignificant toluene removal (about 5%) while all of the samples were photoactive towards the degradation of the model pollutant. Figure 7a shows that the highest degradation after 60 min of irradiation was achieved in the presence of the Ti_90_Mn_10__30V sample (43%). The samples anodized from Ti_90_Mn_10_ alloys at 40 V and 50 V reported similar toluene removal, 28% and 33% respectively. The results displayed in Figure 7b indicate that the manganese content in the alloy inversely affected the photoactivity, the higher the manganese content in the alloy was, the less degradation was achieved. This way, samples prepared from alloys with 5, 10 and 15 wt. % of manganese reached a degradation of 29%, 28%, and 24%, respectively. This was also observed by Xue, Huang, Wang, Wang, Gao, Zhu, and Zou [39] in the dye-mediated photodegradation of MB under visible light in the presence of mesoporous MnO_2_/TiO_2_ nanocomposites. They attributed the lower degradation to the accumulation of MnO_2_ on the surface of TiO_2_ which increased the transfer rate of photogenerated electrons within MnO_2_, overall weakening the effect of improving the photoactivity. The photoactivity of the samples from the series with different water content in the electrolyte was similar between each other (Figure 7c), the highest toluene removal (28%) was accomplished by the sample with 10% of water in the electrolyte. The other two samples exhibited 24% of toluene removal. The kinetic parameters of each photocatalyst are included in Table 2.

The highest initial reaction rate (8.54 ± 0.53 × 10^−2^ μmol·dm^−3^·min^−1^) and reaction rate constant (9.57 ± 0.59 × 10^−3^ min^−1^) were observed for the toluene degradation over the Ti_90_Mn_10__30V sample and they were more than 23 times higher compared with those of pristine TiO_2_ NTs obtained by anodization at 30 V (0.37 ± 0.09 × 10^−2^ μmol·dm^−3^·min^−1^ and 0.42 ± 0.10 × 10^−3^ min^−1^).

**Figure 8:**
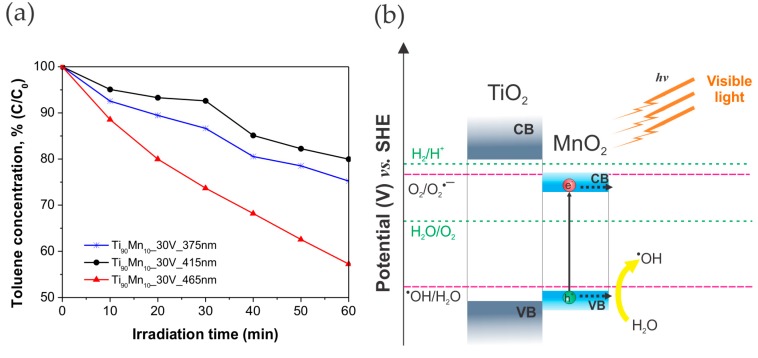
(**a**) Photoactivity of Ti_90_Mn_10__30V sample in gas phase degradation of toluene under different wavelengths of irradiation (λ_max_ = 375, 415, 465 nm) and (**b**) possible excitation mechanism of TiO_2_–MnO_2_ NTs under Vis light irradiation.

As shown in Table 2, the most photoactive sample, Ti_90_Mn_10__30V, was used to analyze the effect of the irradiation wavelength (λ_max_ = 375, 415 and 465 nm) in the same degradation reaction. As evident from Figure 8a, the maximum toluene removal (43%) was reached under 465 nm while under 375 nm (UV light) and 415 nm (25% and 20% of degradation, respectively) the sample was less active. This can be explained by a synergistic effect of MnO_2_ and TiO_2_ in the NT matrix. As it was formerly reported [58], MnO_2_ has lower photoactivity than TiO_2_ under UV light irradiation, and thereby, this narrow-bandgap semiconductor reduced the overall photoactivity of the composite in this wavelength range because of a synergistic effect in the composite. This behavior under UV light was also exposed by Xue, et al. [39] who indicated that the transferring of photoexcited electrons (generated in TiO_2_) within MnO_2_ can correspond to an internal dissipation able to suppress the photocatalytic activity. On the other hand, Xu, et al. [58] reported improved visible light-photoactivity for NTs electrodeposited with MnO_2_. Therefore, we can conclude that the presence of MnO_2_ in TiO_2_ NTs favored the conditions for the degradation of toluene in gas phase under visible light (longer wavelength of irradiation) owing to the ability of MnO_2_ species to absorb visible light irradiation and promote the enhancement of the charge transfer rate.

Additionally, a possible excitation mechanism of TiO_2_–MnO_2_ NTs under Vis light was proposed and diagrammed in Figure 8b. The conduction band and valence band edge values of MnO_2_ were calculated to be 0.57 and 2.34 eV, respectively [34]. Thus, it is likely that photogenerated holes from the valence band (VB) of MnO_2_ could be involved in the formation of hydroxyl radicals (^•^OH), while electrons from the CB of MnO_2_ can participate indirectly in the degradation of toluene, considering that the potential of photogenerated electrons is not high enough to generate other reactive oxygen species, such as O_2_^•−^, H_2_O_2_, and HO_2_^•^ radicals.

## 3. Materials and Methods

### 3.1. Materials

Acetone, isopropanol, and methanol were purchased from P.P.H. “STANLAB” Sp. J. (Lublin, Poland), while ethylene glycol (EG) from CHEMPUR and ammonium fluoride from ACROS ORGANICS. Technical grade Ti foils and Ti–Mn alloys with 5, 10 and 15 wt. % of manganese content were provided by HMW-Hauner Metallische Werkstoffe (Röttenbach, Germany). Deionized (DI) water with conductivity of 0.05 μS was used to prepare all aqueous solutions.

### 3.2. Synthesis of Pristine TiO_2_ and TiO_2_–MnO_2_ Nanotubes

Ti foils as well as Ti–Mn alloys were ultrasonically cleaned in acetone, isopropanol, methanol, and deionized water for 10 min, respectively. Then, foils were dried in an air stream. The anodization processes were carried out at room temperature, in an electrochemical cell consisting of a platinum mesh as counter electrode, and the Ti foils or the Ti–Mn alloy (2.5 cm × 2.5 cm) as working electrode. A reference electrode of Ag/AgCl connected to a digital multimeter (BRYMEN BM857a) was used to control and record information about the actual potential and current on the alloy. The anodization was conducted in an electrolyte composed of EG, water and NH_4_F 0.09 M, during 60 min with a voltage in the range of 30–50 V applied with a programmable DC power supply (MANSON SDP 2603). Three electrolyte solutions with different water content were used (volume ratios of EG:water of 98:2, 95:5 and 90:10) The obtained samples were rinsed with deionized water, sonicated in deionized water (1 min), dried in air (80 °C for 24 h), and calcined (450 °C, heating rate 2 °C/min) for 1 h.

### 3.3. Characterization of Pristine TiO_2_ and TiO_2_–MnO_2_ Nanotubes

The morphology of synthesized pristine TiO_2_ and TiO_2_–MnO_2_ nanotubes was determined by using scanning electron microscopy (SEM, FEI QUANTA 3D FEG, FEI Company, Brno, Czech Republic). Energy-dispersive X-ray spectroscopy (EDX) analysis were performed with a scanning electron microscope (SEM, Zeiss, Leo 1430 VP, Carl Zeiss, Oberkochen, Germany) coupled to an energy-dispersive X-ray fluorescence spectrometer (EDX) Quantax 200 with the XFlash 4010 (Bruker AXS, Karlsruhe, Germany) detector. The crystal structure of the samples was determined from X-ray diffraction patterns recorded in the range of 2θ = 20°–90°, using an X-ray diffractometer (X’Pert Pro, Panalytical, Almelo, The Netherlands) with Cu Kα radiation. The crystallite size was calculated based on the Scherrer formula. Raman spectra were measured with a micro-Raman spectrometer (Senterra, Bruker Optik, Billerica, MA, USA) with a 532 nm excitation laser. The UV-Vis absorbance spectra were registered with the UV-VIS Spectrophotometer, SHIMADZU UV-2600, in the wavelength range of 300–800 nm equipped with an integrating sphere. The baseline was determined with barium sulfate as reference, the scanning speed was 250 nm/min at room temperature. The photoluminescence (PL) spectra were recorded at room temperature with a LS-50B Luminescence Spectrometer equipped with a Xenon discharge lamp, as an excitation source, and a R928 photomultiplier as detector. The excitation radiation (300 nm) was directed on the surface of the samples at an angle of 90°.

### 3.4. Measurement of Photocatalytic Activity

Photocatalytic activity of the as-prepared NTs was analyzed, for the first time, in the purification of air from toluene which was used as a model pollutant. The photodegradation experiments were carried out in a stainless steel reactor of a volume of ca. 35 cm^3^. The reactor included a quartz window, two valves and a septum. The light source consisting of an array of 25 LEDs (λ_max_ = 375, 415 and 465 nm, Optel, Opole, Poland) was located above the sample. The anodized foil was placed at the bottom side of the reactor and it was closed with the quartz window. A gas mixture (toluene, 200 ppmv) was passed through the reactor for 1 min, then the valves were closed and the reactor was kept in dark for 30 min in order to achieve the equilibrium. Before starting the irradiation, a reference toluene sample was taken. The concentration was determined by using a gas chromatograph (TRACE 1300, Thermo Scientific, Waltham, MA, USA), equipped with an ionization flame detector (FID) and an Elite-5 capillary column. The samples (200 μL) were dosed with a gas-tight syringe each 10 min. Irradiation intensity was measured by an optical power meter (HAMAMATSU, C9536-01, Hamamatsu, Japan) and reached 14.7, 14.1 and 14.5 mW/cm^2^ for LEDs with λ_max_ = 375, 415 and 465 nm, respectively.

## 4. Conclusions

The analysis of the effect of applied potential, manganese content in the alloy and water content in the electrolyte on the morphology and visible-light photocatalytic activity of TiO_2_–MnO_2_ NTs obtained from one-step anodic oxidation of Ti–Mn alloys in a fluoride-containing EG-based electrolyte was reported here for the first time. All fabricated samples were described as vertically-oriented, self-organized nanotube arrays with a diameter of 76–115 nm and length of 1–3.4 μm. Diameter and length were directly influenced by the applied voltage while the manganese content led to obtain shorter tubes than those prepared from Ti sheets. The as-prepared TiO_2_–MnO_2_ arrays exhibited improved optical and photocatalytic properties in comparison with those of pristine TiO_2_ NTs. The photoactivity assessment was carried out towards the degradation of toluene (200 ppmv) in gas phase under Vis light irradiation (LEDs, λ_max_ = 465 nm). The highest degradation after 60 min of irradiation corresponded to 43% and the initial reaction rate reached values of 3.79–8.54 × 10^−2^ μmol·dm^−3^·min^−^^1^. A wavelength dependence exploration was performed as well, MnO_2_ modified NTs showed the highest activity under visible light irradiation and therefore, a possible mechanism of excitation was presented. These findings suggest that TiO_2_–MnO_2_ mixed oxide nanotube arrays, activated by low-powered LEDs, could be a promising material for air purification systems. Moreover, the electrochemical approach is a successful way to obtain these highly-organized nanostructures from Ti–Mn alloys. Consequently, the industrially-oriented application of photocatalysis for air treatment using LEDs, as a low-cost and suitable irradiation source, follows the trends of green chemistry and environmentally friendly performance.

## Figures and Tables

**Table 1:** Sample labels, preparation conditions, and selected properties of pristine TiO_2_ and TiO_2_–MnO_2_ nanotubes.

Sample Label	Preparation Parameters	External Diameter (nm)	Tube Length (μm)	Wall Thickness (nm)	Average Crystallite Size (nm)	EDX Analysis			
	Electrolyte, Applied Voltage					Ti (wt. %)	Mn (wt. %)	C (wt. %)	O (wt. %)
Ti_30V	EG 98% (*v*/*v*), H_2_O 2% (*v*/*v*), NH_4_F 0.09 M, 30 V	81 ± 9	1.5 ± 0.1	10 ± 2	33	71.47	0	0.19	28.34
Ti_40V	EG 98% (*v*/*v*), H_2_O 2% (*v*/*v*), NH_4_F 0.09 M, 40 V	100 ± 7	5 ± 0.4	13 ± 2	34	66.73	0	0.03	33.24
Ti_50V	EG 98% (*v*/*v*), H_2_O 2% (*v*/*v*), NH_4_F 0.09 M, 50 V	120 ± 12	16.2 ± 0.2	18 ± 3	38	67.69	0	0.03	32.28
Ti_90_Mn_10__30V	EG 98% (*v*/*v*), H_2_O 2% (*v*/*v*), NH_4_F 0.09 M, 30 V	76 ± 9	1 ± 0.1	8 ± 3	31	76.15	8.91	0.01	14.83
Ti_90_Mn_10__40V	EG 98% (*v*/*v*), H_2_O 2% (*v*/*v*), NH_4_F 0.09 M, 40 V	92 ± 8	1.5 ± 0.1	9 ± 3	32	82.73	7.77	0.01	9.51
Ti_90_Mn_10__50V	EG 98% (*v*/*v*), H_2_O 2% (*v*/*v*), NH_4_F 0.09 M, 50 V	118 ± 4	2.8 ± 0.1	9 ± 2	34	68.79	6.46	0.03	24.72
Ti_85_Mn_15__40V_2%	EG 98% (*v*/*v*), H_2_O 2% (*v*/*v*), NH_4_F 0.09 M, 40 V	94 ± 11	1.3 ± 0.1	9 ± 2	31	77.20	11.14	0.01	11.67
Ti_85_Mn_15__40V_5%	EG 95% (*v*/*v*), H_2_O 5% (*v*/*v*), NH_4_F 0.09 M, 40 V	90 ± 7	1.3 ± 0.1	9 ± 2	35	79.94	12.40	0.01	7.66
Ti_85_Mn_15__40V_10%	EG 90% (*v*/*v*), H_2_O 10% (*v*/*v*), NH_4_F 0.09 M, 40 V	115 ± 8	1.1 ± 0.1	11 ± 2	34	61.76	9.11	1.18	27.95
Ti_95_Mn_5__40V	EG 98% (*v*/*v*), H_2_O 2% (*v*/*v*), NH_4_F 0.09 M, 40 V	94 ± 8	3.4 ± 0.3	9 ± 1	32	70.89	2.10	0.03	27.00

**Table 2:** Initial reaction rate and reaction rate constant for the gas phase degradation of toluene (200 ppmv) under Vis light irradiation (25-LED array, λ_max_ = 465 nm, irradiation intensity = 14.5 mW·cm^−2^) in the presence of pristine TiO_2_ and TiO_2_–MnO_2_ NTs.

Sample Label	Photocatalytic Toluene Degradation	
	Initial Reaction Rate × 10^2^ (μmol·dm^−3^·min^−1^)	Reaction Rate Constant × 10^3^ (min^−1^)
Ti_30V	0.37 ± 0.09	0.42 ± 0.10
Ti_40V	0.43 ± 0.09	0.49 ± 0.10
Ti_50V	0.64 ± 0.04	0.72 ± 0.04
Ti_90_Mn_10__30V	8.54 ± 0.53	9.57 ± 0.59
Ti_90_Mn_10__40V	4.97 ± 0.30	5.57 ± 0.33
Ti_90_Mn_10__50V	6.04 ± 0.08	6.77 ± 0.09
Ti_85_Mn_15__40V_2%	4.18 ± 0.77	4.69 ± 0.87
Ti_85_Mn_15__40V_5%	3.79 ± 0.43	4.24 ± 0.48
Ti_85_Mn_15__40V_10%	5.84 ± 1.61	6.54 ± 1.81
Ti_95_Mn_5__40V	5.76 ± 0.12	6.45 ± 0.14

## Supplementary Files

Supplementary File 1
