# Supplementary material for: Self-Organized TiO2–MnO2 Nanotube Arrays for Efficient Photocatalytic Degradation of Toluene
Source: Molecules. 2017 Mar 31;22(4):564. doi: 10.3390/molecules22040564 (PMC6154631; doi:10.3390/molecules22040564)
Supplement: Supplementary File 1 [file molecules-22-00564-s001.pdf]

List of changes:

Graphical Abstract: Corrected version of graphical abstract was submitted after revision (and it was not skipped during proof preparation.

<table>
<tr><td>Old graphical abstract</td><td>Corrected graphical abstract</td></tr>
<tr><td>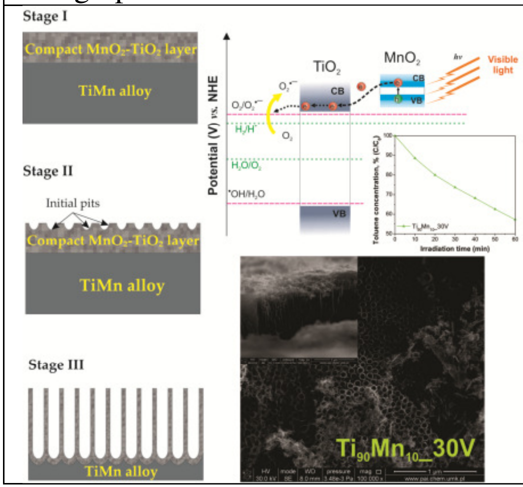
</td><td>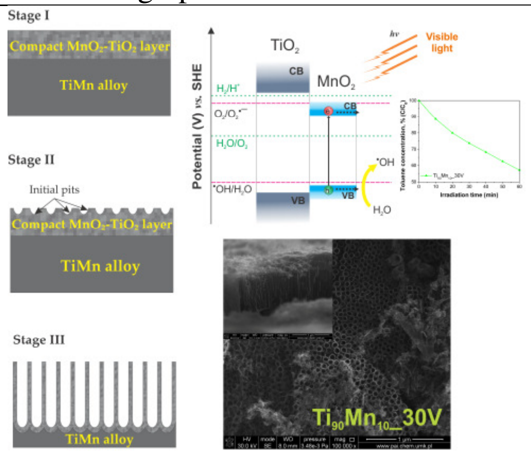
</td></tr>
</table>

Page No	Line No	Old version	Corrected version
1	4	Marek Kobylański	Marek P. Kobylański
1	13	M.K.	M.P.K.
4		**Figure 2.** Proposed growth mechanism of MnO_2–TiO_2 NTs.	**Figure 2.** Proposed growth mechanism of TiO_2–MnO_2 NTs.
5	1	**Table 1.** Sample labels, preparation conditions, and selected properties of pristine TiO_2 and MnO_2–TiO_2 nanotubes.	**Table 1.** Sample labels, preparation conditions, and selected properties of pristine TiO_2 and TiO_2–MnO_2 nanotubes.
10	17 - 26	The morphology of synthesized pristine TiO_2 and TiO_2–MnO_2 nanotubes was determined by using scanning electron microscopy (SEM, FEI QUANTA 3D FEG). Energy-dispersive X-ray spectroscopy (EDX) analysis were performed with a scanning electron microscope (SEM, Zeiss, Leo 1430 VP) coupled to an energy-dispersive X-ray fluorescence spectrometer (EDX) Quantax 200 with the XFlash 4010 (Bruker AXS) detector. The crystal structure of the samples was determined from X-ray diffraction patterns recorded in the range of 2θ = 20°–90°, using an X-ray diffractometer (X'Pert Pro, Panalytical,) with Cu Kα radiation. The crystallite size was calculated	The morphology of synthesized pristine TiO_2 and TiO_2–MnO_2 nanotubes was determined by using scanning electron microscopy (SEM, FEI QUANTA 3D FEG, FEI Company, Brno, Czech Republic). Energy-dispersive X-ray spectroscopy (EDX) analysis were performed with a scanning electron microscope (SEM, Zeiss, Leo 1430 VP, Carl Zeiss, Oberkochen, Germany) coupled to an energy-dispersive X-ray fluorescence spectrometer (EDX) Quantax 200 with the XFlash 4010 (Bruker AXS, Karlsruhe, Germany) detector. The crystal structure of the samples was determined from X-ray diffraction patterns recorded in the range of 2θ = 20°–90°, using an X-ray

		based on the Scherrer formula. Raman spectra were measured with a micro-Raman spectrometer (Senterra, Bruker Optik,)	diffractometer (X'Pert Pro, Panalytical, Almelo, The Netherlands) with Cu Kα radiation. The crystallite size was calculated based on the Scherrer formula. Raman spectra were measured with a micro-Raman spectrometer (Senterra, Bruker Optik, Billerica, MA, USA)
10	39	Optel, Poland	Optel, Opole, Poland
10	44	Thermo Scientific	Thermo Scientific,Waltham, MA, USA
10	47	Hamamatsu City	Hamamatsu City, Japan
11	25	M.K.	M.P.K.
11	26	M.K.	M.P.K.
